# Wind of change: a diurnal skink thermoregulates between cooler set-points and for an increased amount of time in the presence of wind

**DOI:** 10.1242/jeb.244038

**Published:** 2022-03-30

**Authors:** Evelyn Virens, Alison Cree

**Affiliations:** Department of Zoology, University of Otago, PO Box 56, Dunedin 9054, Aotearoa, New Zealand

**Keywords:** *Oligosoma*, Dehydration, Dry-skinned ectotherm, Optimality model, Behavioural thermoregulation

## Abstract

Wind has the potential to dramatically alter the thermal landscape of habitats, and consequently, to affect how ectotherms thermoregulate. However, few studies have directly assessed if wind alters thermoregulation by ectotherms. We compared the thermoregulation of a heliothermic New Zealand skink under three treatments: no wind, wind at 2 m s^−1^ and wind at 6 m s^−1^. We provided captive skinks with housing in which their preferred body temperature was only achievable inside a wind tunnel. During experimental treatments with wind, airflow was generated through the wind tunnel while the maximum available operative temperature remained consistent among treatments. Skinks were able to move in and out of the wind tunnel. Using thermal bio-loggers, we recorded near-continuous skin temperatures of skinks over 90 min. Contrary to our expectations, more skinks tended to thermoregulate in the two wind treatments compared with the treatments without wind (*P*=0.062) and of the skinks that did thermoregulate, those in the two wind treatments thermoregulated for significantly longer than those in the treatment without wind. The set-point temperatures that skinks thermoregulated between became significantly cooler as windspeed increased, despite skinks having access to the same operative temperatures. Overall, our study suggests that wind has the potential to significantly change the temperatures selected by lizards, even when comparable temperatures are available; wind is therefore an important environmental parameter to consider when studying the thermal ecology of ectotherms, including in the context of climate change.

## Abstract (precis)

**Summary:** Under experimental conditions where skinks could raise their body temperatures only by entering a wind tunnel, basking continued but thermoregulatory set-points were lowered in the presence of wind.

## INTRODUCTION

In animals, thermoregulation is the maintenance of body temperatures (*T*_b_) that conform to a mean or variance that is distinct from that of their environment. Thermoregulation increases performance, as *T*_b_ is optimised for biochemical reactions (Angilletta, 2009). While ectotherms are more susceptible to environmental fluctuations in temperature than endotherms, they are often still capable of precise and accurate thermoregulation through behavioural or physiological interactions with their environment (Angilletta, 2009). In ectotherms, precise thermoregulation refers to low variability of *T*_b_ over time, whereas accuracy of thermoregulation (db) refers to the difference between *T*_b_ and the animal's preferred temperature range (or set-point range): the lower the difference, the higher the accuracy (Hertz et al., 1993). As thermoregulation can be energetically expensive and can increase susceptibility to predation, the precision and accuracy with which ectotherms thermoregulate may vary with the associated costs, and in some situations, environmental conditions may preclude thermoregulation for ectotherms altogether (Carter et al., 2010; Sears and Angilletta, 2015).

Wind as an environmental parameter has the potential to affect the accuracy of thermoregulation of ectotherms. Wind modulates the rate of heat exchange between animals and their environments (Porter and Gates, 1969). Additionally, inclusion of wind as a parameter in biophysical models of ectotherms has demonstrated the influence of wind on energy budgets (Kearney and Porter, 2009). However, there is limited knowledge as to how wind affects the thermoregulation of lizards *in situ* and, to our knowledge, no experimental studies have measured the effects of wind on thermoregulation under controlled conditions. Estimates have shown that the effectiveness of lizard thermoregulation (effective regulation being when *T*_b_ is within the set-point range) is constrained in habitats that are more exposed to wind (Ortega et al., 2017). Increased wind has also been associated with lower *T*_b_ in a species of tree iguana (Maia-Carneiro et al., 2012; Gontijo et al., 2018) and with restricted ability to elevate *T*_b_ above air temperature (*T*_a_) in a New Zealand skink (Hare et al., 2009).

Although *T*_a_ is predicted to increase in most environments over the next century (Pachauri et al., 2014), changes in wind patterns are expected to be more variable. Wind velocity is predicted to change (either increase or decrease) at both global (McInnes et al., 2011) and regional (Pryor and Barthelmie, 2010) scales. Also, there is often relatively high uncertainty associated with projections of future wind conditions compared with other climate variables (Pryor et al., 2005; Pryor and Barthelmie, 2011; Solaun and Cerdá, 2019). Given this variation and relative uncertainty, it is important to determine how wind might affect the thermoregulation of lizards that may already be vulnerable to rising *T*_a_.

In this experiment we examined how the velocity of artificial wind affected the likelihood that skinks would thermoregulate in comparison to windless conditions. Additionally, for skinks that did thermoregulate in the presence of wind, we compared thermoregulation between treatments with wind and those without. Our study species, the diurnal McCann's skink [*Oligosoma maccanni* (Patterson and Daugherty 1990)] from New Zealand, was chosen for this experiment as it has been extensively studied previously in laboratory experiments and its range includes sub-alpine environments that are subject to frequent strong winds. McCann's skink is an actively foraging, heliothermic and saxicolous species. We used miniaturised thermal bio-loggers (Virens and Cree, 2018) to obtain near-continuous measurements of skink skin temperature (*T*_sk_) under three windspeeds while keeping *T*_a_ and substrate temperature constant. We hypothesised that skinks would be less likely to thermoregulate in the presence of wind and that thermoregulation would become less accurate as windspeed increased.

## MATERIALS AND METHODS

We collected 30 adult McCann's skinks (mean body mass=3.32 g) by hand, over 2 days, from schist tors in grazed pasture and mixed tussock grassland. The collection site is in Eastern Otago in New Zealand (−45°4′S, 170°4′E) and is sub-alpine: 680–700 masl. The use of skinks was conducted with authorisations from the New Zealand Department of Conservation and the University of Otago Animal Ethics Committee and followed consultation with Kāti Huirapa Rūnaka ki Puketeraki.

Two skinks were collected in early January 2018 for a pilot study to determine the wind speeds to be used. In early February 2018, 28 skinks (14 non-pregnant females and 14 males) were collected for the main experiment. Following capture, skinks were transported to the University of Otago and, on arrival, ectoparasitic mites were removed by smothering with sunflower oil (Hare et al., 2010). Skinks were then moved to a temperature-controlled room that simulated summer conditions at the collection site: 16°C by day, 13°C by night, with a photoperiod (14 h light:10 h dark) that included 2 h dusk and dawn ramps. Relative humidity recorded at the beginning of each test ranged from 57 to 64%.

**Fig. 1.**
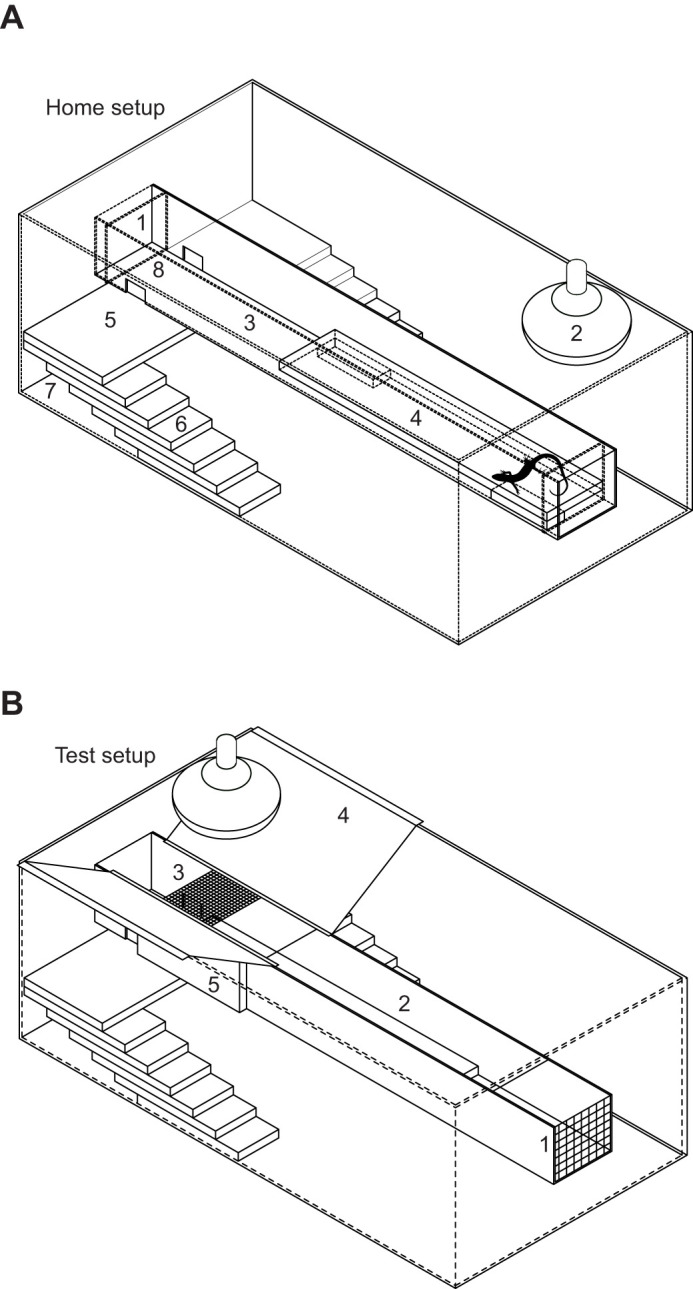
**Skink housing conditions between and during tests.** (A) Skink housing between tests. The primary containers were plastic tubs (480×270×107 mm) with a wire mesh lid. Each container had 65×55 mm apertures (1) cut into the centre of the longitudinal sides. A heat lamp (30 W or 40 W incandescent light bulb) (2), was suspended above a U-shaped white PVC bridge (3) supported between the two apertures. Both ends of the bridge were closed with pieces of 65×55×30 mm polystyrene foam (not shown). A ceramic tile (300×65×5 mm) was placed in the bridge beneath the heat lamp (4). This basking tile was supported on top of two smaller tiles (65×10×5 mm) which created a warm retreat area beneath. Beneath the bridge a large ceramic tile (5; 200×100×5 mm) was placed on top of two stacks of six smaller tiles (6; 100×40×5 mm) that formed stairways. This created two cool retreat areas beneath the stair stacks (7) that remained at ambient temperature (*T*_a_). Two access holes (30×20 mm) were cut into both sides of the bridge where it sat on top of the larger tile (8). Skinks accessed the basking area and warm retreat area in the bridge by climbing one of the stairways and passing through the access hole. A plastic water dish and a plastic dish containing damp sphagnum moss (not shown) were placed at the base of the container. (B) Skink housing during testing. The polystyrene blocks were removed from the ends of the wind tunnel and replaced with a piece of stainless-steel wire mesh (mesh size=5×5 mm, gauge=0.5 mm) which allowed airflow through the wind tunnel while preventing the skink from escaping (1). The top of the bridge was covered with four abutting sections of 10-mm-thick transparent Plexiglass to create a wind tunnel (2). A 65×55 mm piece of wire mesh (mesh size=5.5×5.5 mm, gauge=0.8 mm) covered the space directly underneath the heat lamp to allow heat through to the basking tile (3). Two baffles (4) made of stainless-steel plate (20 ×12 mm, 0.5 mm sheet thickness) were used to cover the space between the edge of the wind tunnel and the side of the housing container so that the heat lamp did not warm the base of the housing container. Additional stainless-steel plate baffles (102×51×11 mm, 0.5 mm sheet thickness) were placed along the sides of the wind tunnel to minimise heat transfer through the walls of the wind tunnel (5).

Skinks were housed individually in plastic containers with wire mesh lids. Each skink had access to a warm basking tile and warm retreat area in a bridge suspended above the container floor, as well as a cool retreat area at the base of the container (Fig. 1A). The basking area was heated by 40 or 60 W incandescent light bulbs, which were adjusted using a dimmer control so that warmest area reached 32±1°C within the first hour of being switched on. The base of the container, including the cool retreat areas, remained at ambient temperature (15–18°C by day). We used a handheld infrared thermometer (Fluke 568, Fluke Corporation, Everette, USA) to verify that the maximum cage temperatures were not above 33°C frequently during the period of captivity. Basking lamps and an overhead ultraviolet light (Arcadia D3 reptile lamp, Croydon, UK) were turned on following the morning ramping period for 6 h day^−1^. A tray of damp sphagnum moss and a small water dish were provided in the container. With this housing, all skinks consistently entered the bridge to bask within 4 days of capture and before testing began. Once a week, each skink was fed one mealworm larva (*Tenebrio molitor*) and one cricket (*Teleogryllus commodus*) dusted with vitamin supplement, and with ∼5 g fruit puree (mango, peach or papaya). The two skinks used for pilot tests were returned to their field collection site within 7 days of capture whereas the skinks used in the main experiment were returned within 66–67 days of capture. All skinks were in good condition and had an average increase in mass of 0.17 g when returned.

To test the effects of wind on thermoregulation, we measured *T*_sk_ for each skink, over the course of three 90 min tests. During the tests, the bridge was fitted with a Plexiglass cover to create a wind tunnel. Skinks could choose either to bask in the wind tunnel, or to avoid the wind tunnel and basking lamp by moving to the base of the container. Each skink was tested three times, once each for three different wind speeds: 0 m s^−1^, 2 m s^−1^ and 6 m s^−1^. The skinks remained in their housing containers during tests, which were modified to facilitate the treatment (Fig. 1B). During the tests, skinks were able to move freely between the heated wind tunnel and the base of the container, which remained at ambient temperature. To prevent skinks from avoiding wind by entering the warm retreat area, the small tiles that supported the basking tile in the wind tunnel were removed, so that the basking tile sat against the base of the wind tunnel. The maximum operative temperature available to the skinks during each treatment was 40°C. This was achieved by increasing the amount of radiant heat from a 375 W incandescent heat-lamp as the wind level increased. Operative temperatures were determined for each treatment using hollow copper models containing a plastic collar that suspended a thermocouple wire. These models had been previously calibrated to accurately predict the operative *T*_b_ of McCann's skink (Hare et al., 2009). During the tests, operative temperatures of 40°C were maintained through slight adjustments to the output of the heat-lamp with a dimmer control. Operative temperatures measured when the copper model was placed directly beneath the heat-lamp were equal to (±0.5°C) the temperature measured by a thermocouple fixed with a piece of masking tape (20 mm^2^) to the tile directly beneath the heat-lamp; therefore, the latter method was used to monitor the maximum available operative temperature during the tests. An operative temperature of 40°C for the skinks produced a maximum surface temperature of 35°C for the basking tile (when measured with an infrared thermometer). Therefore, the temperature of the basking tile ranged from 35°C directly under the heat lamp, to 18°C at the end furthest from the heat-lamp (Fig. S1, Table S1).

Wind was generated through the wind tunnel with two 80 mm computer fans, either Noctua NF-A8 PWM (Noctua, Vienna, Austria) for 2 m s^−1^ treatments or Delta TFB0812UHE (Delta electronics Inc., Taipei, Taiwan) for 6 m s^−1^ treatments. One fan moved air into the tunnel and the other drew the air out. The speed of each fan was controlled independently by a Noctua NA-FCI control unit (Noctua, Vienna, Austria). The fans were attached to two 90 deg PVC elbows which were connected to two lengths of circular (80 mm internal diameter) PVC pipe. These pipes were linked to the wind tunnel by two PVC square-to-round sockets. The use of 90 deg pipe elbows meant that skinks could not see the fans while inside the wind tunnel; however, the elbows also created turbulence. To compensate for this, the leading pipe connecting the elbow to the wind tunnel was 500 mm in length to facilitate stabilization of the airflow.

To select the windspeed for the 2 m s^−1^ and 6 m s^−1^ treatment groups, we needed first to determine the upper value that skinks would voluntarily expose themselves to when attempting to bask. Two skinks were used in three 30 min pilot tests in which we aimed to determine if skinks would thermoregulate at wind speeds of 6 m s^−1^, 8 m s^−1^ and 10 m s^−1^. Both skinks attempted to bask at all three wind speeds. However, both 10 m s^−1^ treatments were halted early (within 3 min) as the skinks were unable to maintain their basking position and were blown down the wind tunnel (though unharmed). Both skinks also basked in the 8 m s^−1^ treatments, but movements were laboured. Skinks were able to move normally during the 6 m s^−1^ treatment. We therefore chose windspeeds of 2 m s^−1^ and 6 m s^−1^ for treatments. At our study site, operative temperatures for McCann's skinks in basking locations can reach ≥40°C when windspeeds (recorded at a height of 10 m) reach over 10 m s^−1^. Therefore, we consider these treatments to be ecologically relevant.

Thereafter, all tests carried out on each day used the same treatment. Fan speeds were calibrated each day by connecting a dummy housing container to the wind tubes and placing an anemometer (Kestrel 1000 handheld wind meter, KestrelMeters, Pennsylvania, USA) into three positions in the wind tunnel by removing sections of the tunnel's Plexiglass cover. By taking measurements at multiple points along the tunnel we ensured that wind was as consistent as possible along the tunnel's length. Wind was adjusted by changing the speed of each fan independently as required. During the 0 m s^−1^ treatment we operated fans disconnected from the wind tunnel close to the container so that the levels of noise and vibration were similar for each treatment.

Skinks first underwent the experimental procedure after 27–77 days in captivity (mean=52 days) and were randomly grouped into three cohorts (*n*=9, 9, 10). Cohorts were cycled so that skinks always received at least two (but up to 19 with a mean of 7) rest days between tests. Each day, fan speeds were established for one of the treatment levels and up to four skinks previously untested at that treatment level were chosen at random from the same cohort until no choices remained for the final four skinks. Effects from the order of treatments were minimised by this approach as the order varied among skinks within cohorts. Tests took place sequentially, beginning at 09:00 h, 10:30 h, 12:00 h and 13:30 h. Cohorts were denied basking opportunity on days when skinks from that cohort were used in tests. Food was withheld for the entire cohort for 2 days before a group of four skinks from a cohort underwent testing.

**Fig. 2.**
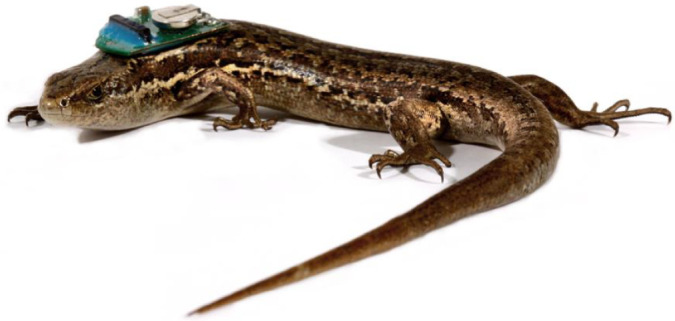
**An adult McCann's skink (*Oligosoma maccanni*) with a thermal bio-logger (0.3 g) attached to the dorsum just behind the head with double-sided cellophane tape.** The bio-logger's mass ranged from 6–10% of skink body mass across all skinks tested (photo credit: Ken Miller).

Skinks were fitted with a thermal bio-logger (Virens and Cree, 2018) 60 min before being tested. Bio-loggers were attached to the skin dorsally behind the head using 12 mm^2^ of double-sided cellophane tape (Sellotape, Winsford, UK; Fig. 2); attachments weighed 0.3 g (6–10% of the skink's body mass) and measured 14 mm×11 mm×2 mm. Prior to attachment the bio-loggers were programmed to record temperature at 2 s intervals for 90 min with a 60 min delay so that they would begin recording data at the beginning of the test. The resolution was set at 0.0625°C. In nearly all instances, skinks were already in the wind tunnel before the test (presumably waiting for the basking lamp to turn on); the loggers could then be placed on the dorsum without handling the skink. In cases where the skink was handled to attach the bio-logger the skink was returned to the wind tunnel at the end furthest from the heat lamp. In all cases, the skinks were already in the bridge section of the housing at the beginning of the test and remained in place while the housing was (carefully) modified. During the tests the maximum operative temperature (measured by the thermocouple taped to the basking tile) was maintained at 40°C and the skink was observed via a video feed from behind a screen.

At the end of each test, the skink's housing was then returned to its normal orientation. At this time, we measured the cloacal temperature of 14 skinks that had previously been thermoregulating (i.e. in the wind tunnel and not in the base of the housing, including a mix of skinks that were both warming and cooling) with a thermocouple to calibrate with the bio-logger temperature before removing the logger. A small amount of acetone was applied to the edge of the bio-logger with a cotton bud to help remove it from the skink. Temperatures recorded by the bio-loggers (*n*=14) were highly correlated with (*R*=0.98, *P*=<0.01) but slightly cooler than cloacal temperatures (measured with a thermocouple) by a mean of 1.32°C (±0.247; Fig. S2). Skinks were allowed normal basking opportunity the following day. The bio-logger's battery was then recharged using a custom-made charger that supplied the battery with a 3.0 V charge current. Any batteries that did not charge to 2.8 V after 8 h of charging were discarded and replaced with a new battery. Six bio-loggers were used in total; the number of times each was reused ranged from once to 21 times (mean 14±2.7).

**Fig. 3.**
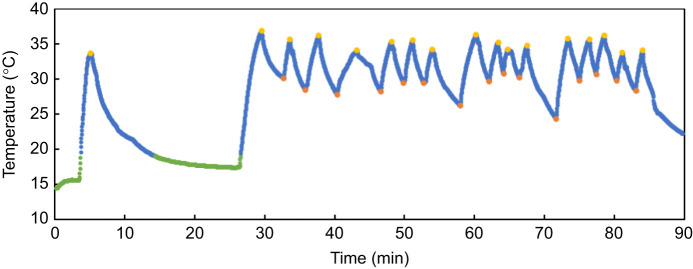
**A representative trace of skink skin temperature (*T*_sk_).** Temperature data were recorded using a bio-logger attached to a McCann's skink during a 0 m s^−1^ test. Blue data points have been labelled as sections where the skink was thermoregulating, having achieved a *T*_sk_ above 18°C. Green data points are those labelled as non-thermoregulating periods, where *T*_sk_ is below 18°C (ambient temperature, *T*_a_). Yellow dots are upper set-point labels and orange dots are lower set-point labels.

Temperature data were downloaded from each bio-logger immediately after each test. We wrote a script in Python (Python Software Foundation, www.python.org) to automatically annotate the temperature traces for analysis. For each trace, temperatures recorded while the skink was in the wind tunnel (above *T*_a_ in the base of the housing container) were labelled as thermoregulating sections, whereas temperatures matching ambient air were labelled as non-thermoregulating sections. An upper set-point label was applied to the datapoint preceding a switch in the direction of temperature change from warming to cooling. Lower set-point labels were applied to the datapoint preceding a switch in the direction of temperature change from cooling to warming. To prevent oversaturating the trace with set-point labels, a sensitivity of 2°C was applied to the labelling process. Therefore, the script would only recognise that the direction of temperature change had reversed once the temperature was ≥2°C below the warmest value of the current warming section or ≥2°C above the coolest value of the current cooling section. All annotated temperature traces were visualised to ensure that the software had performed as expected (Fig. 3).

**Fig. 4.**
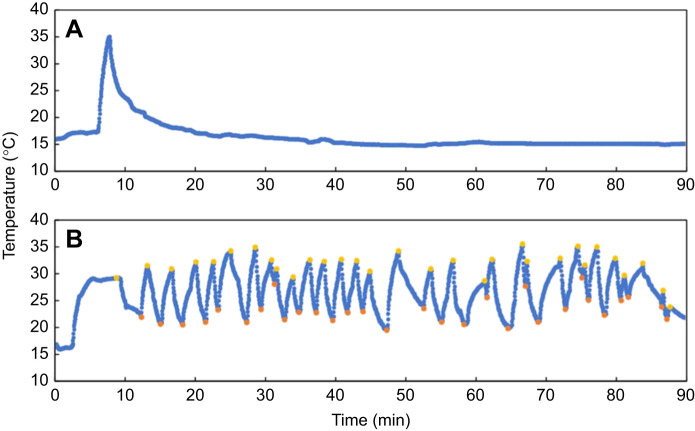
***T_sk_* traces from a non-thermoregulating and a thermoregulating McCann's skink (*O. maccanni*).** (A) Non-thermoregulating skink. (B) Thermoregulating skink. Yellow and orange annotations for B represent upper and lower set-points, respectively.

For each test, skinks were classified as either thermoregulating or non-thermoregulating (Fig. 4). Skinks that exhibited more than two changes in the direction of temperature change (i.e. shuttles) while in the wind tunnel were classified as thermoregulators and these traces were included in the analysis of thermoregulation. Skinks that left the wind tunnel either immediately, or after achieving a single upper set-point, were classed as non-thermoregulators and no further analysis was carried out on these temperature traces.

In describing the thermal characteristics of each temperature profile, we use the term thermal passivity range (TPR, after Kingsbury, 1993) to describe the difference between the mean of the upper set-points and the mean of the lower set-points of each skink. This is to avoid confusion with the term ‘set-point range’, which usually refers to the range of preferred *T*_b_ determined for a species or population measured under controlled conditions on a thermal gradient (Hertz et al., 1993). For each annotated temperature trace from thermoregulating skinks, we calculated seven parameters of thermoregulation: the mean *T*_sk_ for the whole test, the means of the upper set-points and lower set-points, the TPR, the global maximum for the test (*T*_max_), the percentage of the test time that the skink spent thermoregulating in the wind tunnel and the number of shuttles between set-points. To synthesise these parameters we also estimate the accuracy of thermoregulation of skinks in each treatment using the method established by Hertz et al. (1993). For each test we calculated the accuracy of thermoregulation (db) as the mean absolute deviation of all *T*_sk_ measurements (both thermoregulating and non-thermoregulating sections), from the mean TPR of skinks from the thermoregulating sections of the 0 m s^−1^ treatment.

Data analysis was performed using the R programming language (https://www.R-project.org/). Distributions and residuals of the data were visualised to ensure that the assumptions of a normal distribution were met. To determine the relative effects of wind treatment on whether skinks did or did not thermoregulate (as a response variable with a binomial distribution), we used a generalised linear mixed-effects model, using the *glmer* function in the package lme4 (https://CRAN.R-project.org/package=lme4). Wind treatment was a fixed effect and skink identity a random effect. To determine the effects of the experimental treatment on the seven thermoregulation variables and mean absolute variation, we used linear mixed-effect models fitted using the ‘lmer’ function in lme4. Treatment, sex, number of days spent in captivity and mass were included in the models as fixed effects and skink identity as a random effect. We used the ‘Anova’ function in the package car (https://CRAN.R-project.org/package=car) to calculate *P-*values for both the generalised linear mixed-effects model and linear mixed-effect models. This function uses a type II Wald Chi^2^ test to generate *P*-values. Pseudo *R*^2^ values were calculated using the ‘r.squaredGLMM’ function from the MuMIn package (https://CRAN.R-project.org/package=MuMIn). This function calculates revised marginal and conditional *R*^2^ values after Nakagawa et al. (2017). To compare significantly different treatments, we used the ‘glht’ function in the package multcomp (https://CRAN.R-project.org/package=multcomp; Hothorn et al., 2008) to perform a Tukey multiple-comparison test and calculate pairwise *P-*values adjusted using the Holm method (Holm, 1979).

## RESULTS

Skin temperature data were recorded at 2 s intervals from all 28 skinks for each of the three treatments. For 78 tests, data traces between 87 and the intended 90 min were recorded. Six tests (4 from the 6 m s^−1^ treatment, and 1 each from the 2 m s^−1^ and 0 m s^−1^ treatments) ran for only between 50 and 79 min because of skinks escaping. Thereafter, slight adjustments were made: the moss dish was moved beneath the cool retreat area (to prevent skinks gaining extra height to climb the walls) and the metal baffles were secured in place with masking tape. A single test with spurious data was excluded from analyses: upper and lower set-points began to gradually increase until the global maximum for the test was 41°C (implausible as this value approximates the critical thermal maximum of McCann's skink; Virens and Cree, 2019). The drift was probably caused by low voltage of the bio-logger battery.

The ratios of thermoregulators to non-thermoregulators in the three treatments (0, 2 and 6 m s^−1^) respectively were 17:11, 23:5 and 23:4. Non-thermoregulating skinks raised their *T*_sk_ to a single upper set-point within the first 30 min of the test, then left the wind tunnel and did not rise in temperature again for the remainder of the test. A single non-thermoregulating skink in the 0 m s^−1^ treatment remained at the cold end of the wind tunnel for the entire test. In most tests where skinks thermoregulated (50 of 63 tests), skinks remained in the wind tunnel for a single bout of thermoregulation. There were 12 tests where skinks left the wind tunnel between two bouts of thermoregulation (4, 5 and 3 in 0, 2 and 6 m s^−1^ treatments, respectively), and two tests where skinks exhibited three separate bouts of thermoregulation leaving the wind tunnel between each bout (both in the 2 m s^−1^ treatment). The difference among the three treatments in whether or not skinks thermoregulated was not quite significant (*P*=0.062), although there was a trend for more skinks to thermoregulate in the 2 m s^−1^ and 6 m s^−1^ treatments (Table 1A). The difference between conditional and marginal *R*^2^ values produced by the GLM suggests that skink identity explains some of the observed variance (Table 1B). In other words, there was a trend for the presence of thermoregulation of individual skinks to be the same across the three treatments.

**Table 1.**
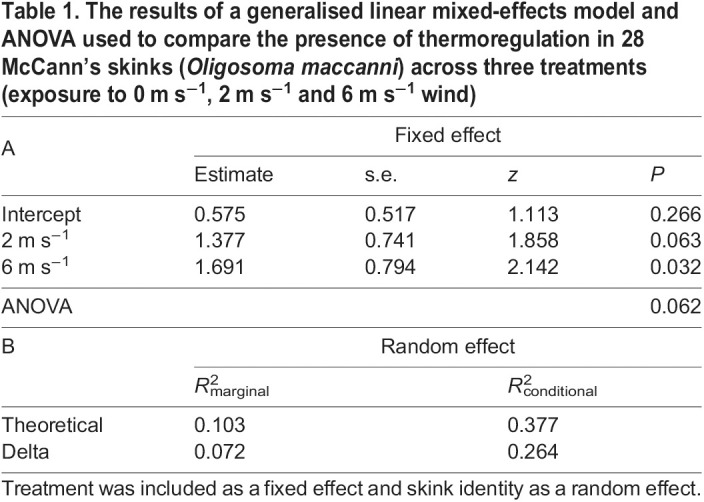
The results of a generalised linear mixed-effects model and ANOVA used to compare the presence of thermoregulation in 28 McCann's skinks (*Oligosoma maccanni*) across three treatments (exposure to 0 m s^−1^, 2 m s^−1^ and 6 m s^−1^ wind)

**Fig. 5.**
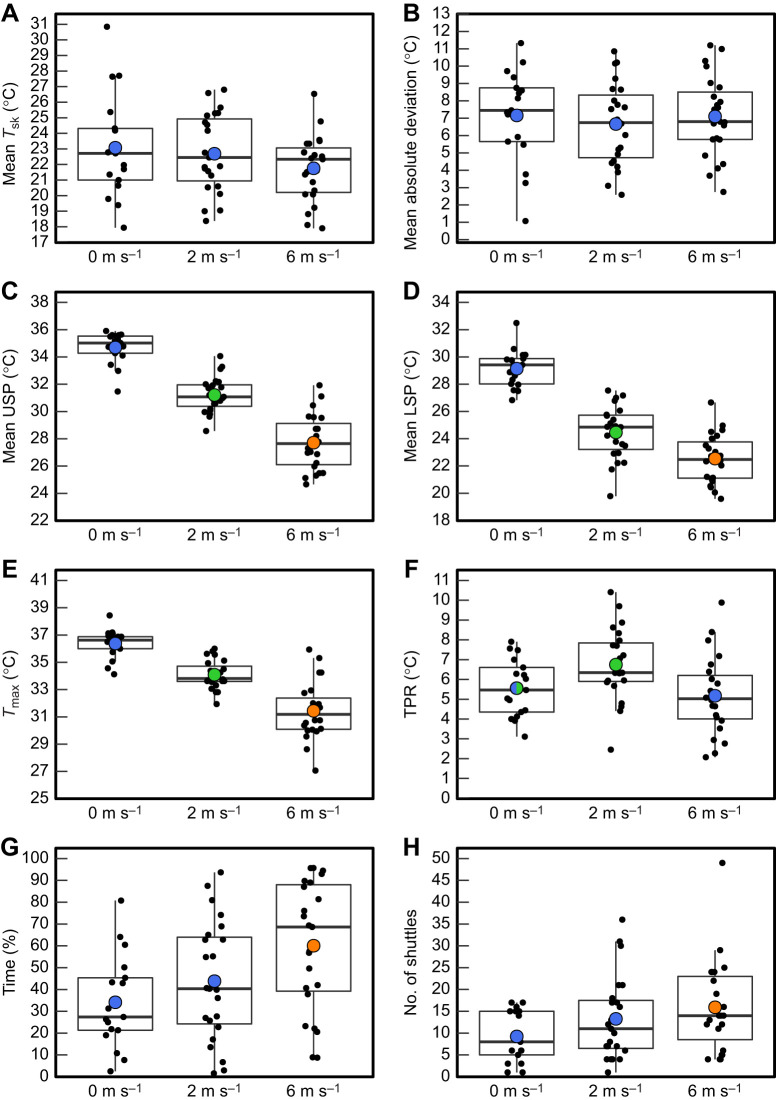
**Boxplots of seven thermoregulation parameters and deviation of skin temperature from a set-point range for McCann's skinks (*O. maccanni*) under three wind treatments: 0 m s^−1^, 2 m s^−1^ and 6 m s^−1^ (*n*=17, 23, 23).** (A) Mean *T*_sk_. (B) Mean absolute deviation of *T*_sk_ from set-point range. (C) Mean upper set-point. (D) Mean lower set-point. (E) Global maximum temperature. (F) Thermal passivity range. (G) Time spent thermoregulating. (H) Number of shuttles. Means are shown as coloured circles where different colours represent means that differ significantly (*P*<0.05). Boxplots show the extent of the maximum and minimum values, the first quartile, median and third quartile. Individual datapoints are shown as black points with horizontal jitter to reveal overlaid points.

The mean *T*_sk_ temperatures (for the entire period of each test) of the skinks that thermoregulated did not significantly differ among treatment groups and the overall mean was 22.5±0.33°C (Fig. 5A). The mean absolute deviation of *T*_sk_ for the whole test was not statistically different among treatments (Fig. 5B). The remaining eight thermoregulation parameters that were measured differed significantly among wind treatments. Mean upper set-point, mean lower set-point and *T*_max_ were all highest in the 0 m s^−1^ treatment, the temperature of all three parameters decreased as wind increased (Tables 2–4) and all three treatment groups were significantly different from each other (Fig. 5C,D,E). Skinks in the 6 m s^−1^ treatment spent significantly more time thermoregulating than in the 0 and 2 m s^−1^ treatments (Fig. 5H). The number of shuttles was significantly higher in the 6 m s^−1^ treatment when compared to the 0 m s^−1^ treatment; however, the number of shuttles that occurred at the 2 m s^−1^ treatment was not statistically different to the other two treatments (Fig. 5H). The broadest TPR occurred during the 2 m s^−1^ treatment (Fig. 5F). The TPR of skinks under the 6 m s^−1^ treatment was significantly narrower than at 2 m s^−1^. Differences between the marginal and conditional *R^2^* values suggest that skink identity accounted for some of the observed variance in the statistical models for TPR, number of shuttles, time spent and thermoregulating (Table 3). Sex, days in captivity and mass did not have a significant effect for any of parameters considered, with the exception that days in captivity was positively correlated with mean upper set-point and *T*_max_.

**Table 2.**
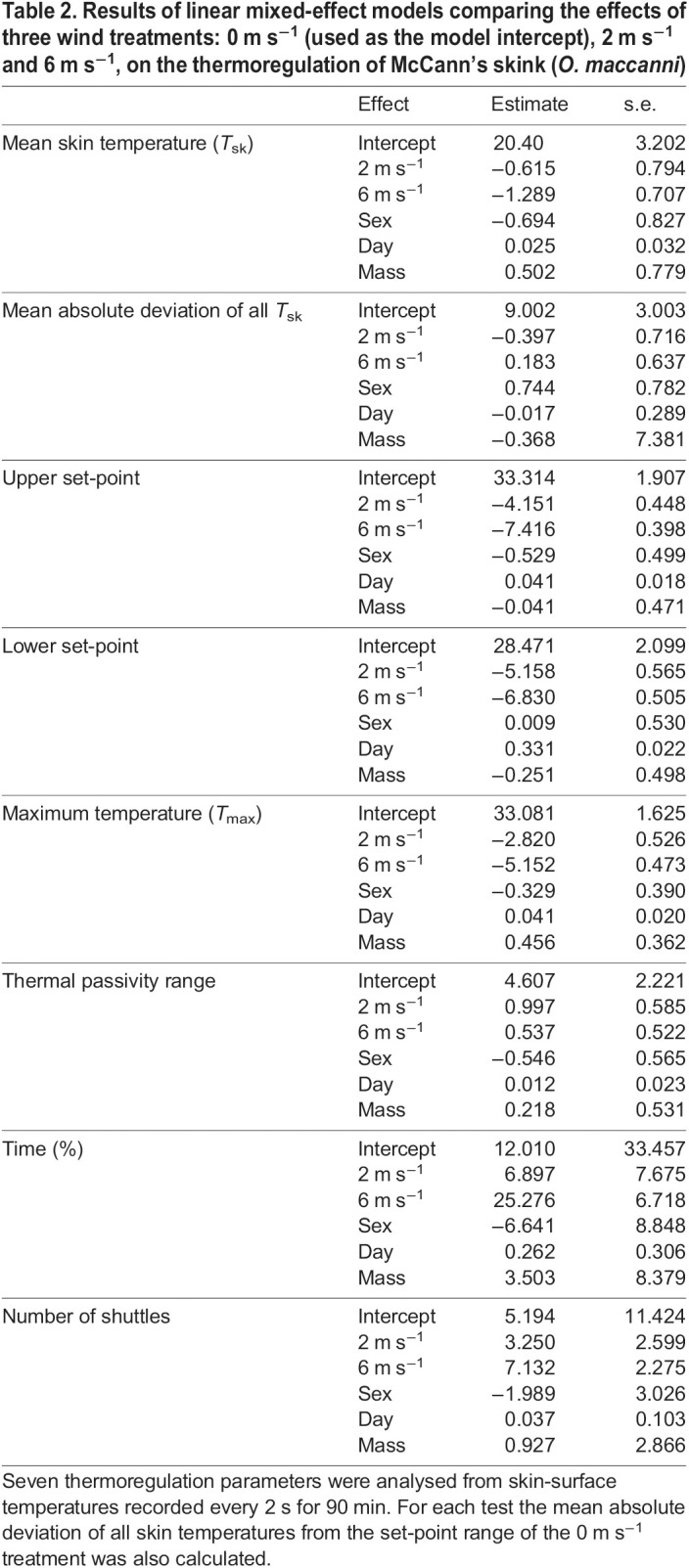
Results of linear mixed-effect models comparing the effects of three wind treatments: 0 m s^−1^ (used as the model intercept), 2 m s^−1^ and 6 m s^−1^, on the thermoregulation of McCann's skink (*O. maccanni*)

**Table 3.**
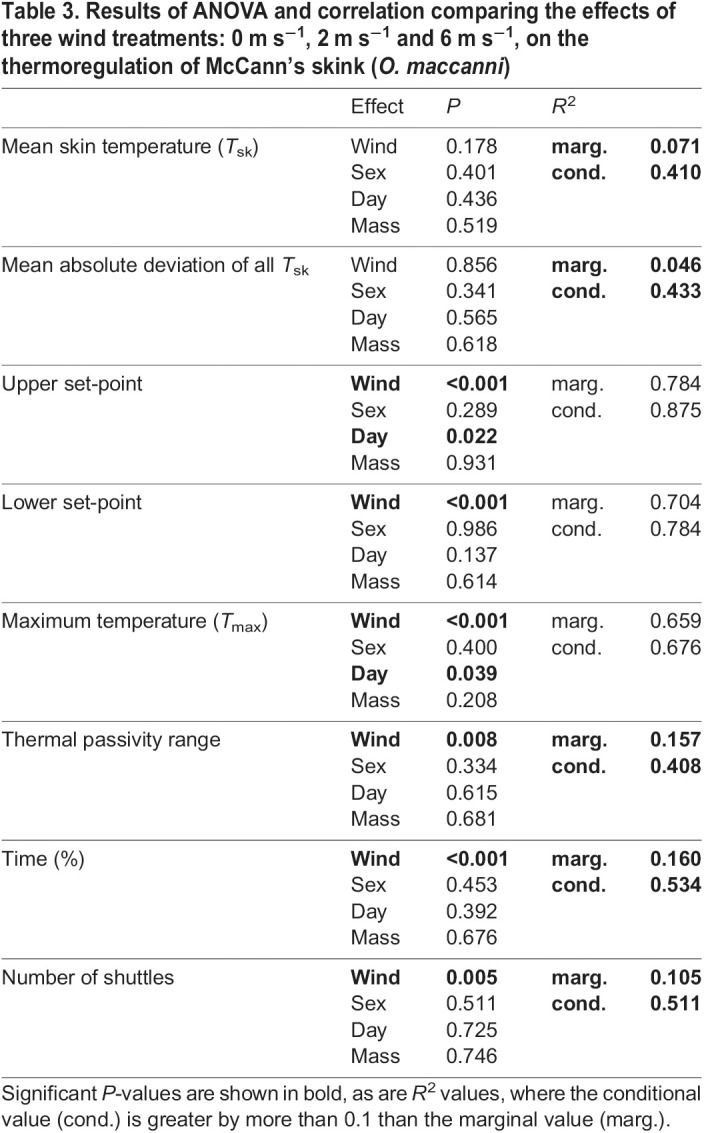
Results of ANOVA and correlation comparing the effects of three wind treatments: 0 m s^−1^, 2 m s^−1^ and 6 m s^−1^, on the thermoregulation of McCann's skink (*O. maccanni*)

**Table 4.**
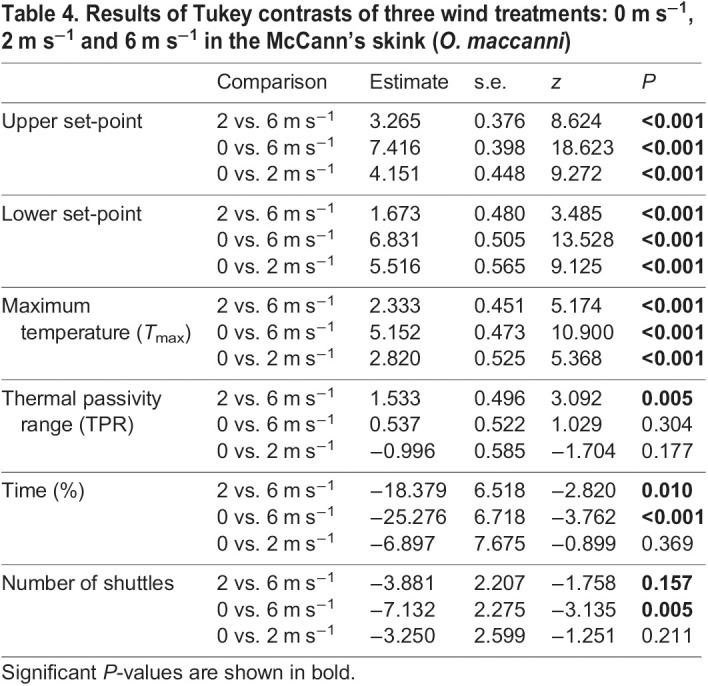
Results of Tukey contrasts of three wind treatments: 0 m s^−1^, 2 m s^−1^ and 6 m s^−1^ in the McCann's skink (*O. maccanni*)

## DISCUSSION

Our study provides the first detailed analysis of thermoregulatory changes in a lizard in response to wind. Under laboratory conditions, McCann's skinks altered their thermoregulatory strategies when exposed to wind. Skinks were at least as likely to bask, and when they did so they thermoregulated between cooler upper and lower set-points as wind increased. As the wind level increased, skinks spent proportionally more time thermoregulating; however, this trend was significant only at 6 m s^−1^. Additionally, the recent miniaturisation of the bio-logger used here (Virens and Cree, 2018) allowed near-continuous temperatures to be measured, which are rarely reported for animals as small as McCann's skink (mean 3.32 g).

In dry-skinned ectotherms, thermoregulation can be affected by physiological factors including reproductive and digestive states, hydration, and metabolic and immune stressors (Black et al., 2019). Changes in thermoregulatory strategy have also been linked to the often-complex costs of thermoregulating under different ecological conditions (Angilletta, 2009). The optimality model of thermoregulation assumes that under certain conditions, the ecological or physiological costs of thermoregulation can outweigh the benefits (Huey and Slatkin, 1976). The model predicts that thermoregulatory strategies will change alongside changes in the costs and benefits associated with thermoregulation (Huey and Slatkin, 1976). Different thermoregulatory strategies then influence how body temperatures relate to available operative temperatures (Angilletta, 2009). According to the optimality model, therefore, the different thermoregulatory strategies that our skinks employed among treatments must reflect the costs (or perceived costs under controlled conditions) of being exposed to wind, as the range and distribution of operative temperatures available to the skinks differed minimally between treatments (Fig. S1). The question then becomes what are the ecological costs of thermoregulating in the wind?

In both wind treatments, McCann's skinks thermoregulated between cooler set-points and in the 6 m s^−1^ treatment spent longer periods actively thermoregulating than under the windless treatment. Previous studies have also shown that lizards selected cooler temperatures when the costs of thermoregulating increased, though not specifically by wind. For example, the small, heliothermic iguanid *Tropidurus torquatus* selected cooler upper and lower set-points when the cost of locomotion was increased (Leirião et al., 2019). However, in *T. torquatus*, overall mean temperatures also decreased as cost increased, whereas we observed similar mean temperatures among treatments. The cost of locomotion may potentially be increased by wind; however, if so, the amount of movement should also be decreased whereas we observed increased movement (i.e. more shuttling). Thermoregulation with decreased movement was observed in bearded dragons (*Pogona vitticeps*) under hypoxic conditions; the number of shuttles decreased as hypoxia increased owing to the increased cost of movement (Cadena and Tattersall, 2009).

Perhaps of most relevance to our results, hydration state has been shown to affect thermal preferences in lizards. The lizard *Zootoca vivipara* thermoregulated between cooler set-points and had a lower mean *T*_b_ when water was withheld for a day (Le Galliard et al., 2021). Dehydration lowered the mean *T*_b_ and preferred body temperatures for four species of wall lizard (*Podarcis*), resulting in left-skewed body temperature distributions (Sannolo and Carretero, 2019). Similarly, lower modal *T*_b_ has been reported for the lizard *Sceloporus undulatus* when dehydrated (Crowley, 1987) and left-skewed body temperature distributions have been reported for dehydrated Western tiger snakes (*Notechis scutatus*) compared with hydrated individuals (Ladyman and Bradshaw, 2003). It has been suggested that selecting lower *T*_b_ in response to dehydration in dry-skinned ectotherms is an adaptive response; lowering *T*_b_ reduces the rate of metabolism which in turn limits metabolic water loss in an attempt to conserve water (Black et al., 2019). In our study, we assume skinks to have been equally well-hydrated at the start of all tests; they also had access to two, familiar sources of water during the tests. Furthermore, patterns of thermoregulation were largely consistent throughout each test. For example, set-points did not begin higher and become cooler as the test progressed, despite the potentially dehydrating effect of wind. Therefore, we suggest that McCann's skinks that were exposed to wind may have thermoregulated over a cooler temperature range in anticipation of becoming dehydrated. In other words, we suggest that the risk of an imminent reduction in hydration state (rather than actual dehydration) offers the most compelling explanation for the differences in thermoregulation observed here. However, as we did not measure evaporative water loss, we cannot rule out the possibility that some dehydration had been voluntarily accepted by skinks by the end of the test. Future studies should attempt to distinguish the roles of a risk to future hydration state and actual dehydration (voluntary or otherwise) in assessing thermoregulatory responses to wind.

Thermoregulation of skinks in the 0 m s^−1^ treatment represents thermoregulation with the least associated costs. The mean TPR of 29.1–34.7°C is analogous to a set-point range or preferred temperature range of McCann's skink under the conditions of our experiment and allowed us to calculate the deviation of body temperatures from this range (mean absolute deviation). This range for TPR is a few degrees higher than previously estimated set-point ranges (as cloacal temperatures for skinks on a thermal gradient) for McCann's skink (24.6–30.0°C for non-pregnant females; Thierry et al., 2009) and 26.4–30.2°C for pregnant females (Hare et al., 2009) but comparable with near continuous temperature measurements of wild skinks using the same bio-loggers (Cree et al., 2021). Our current results may have greater accuracy as near-continuous measurements have been shown to provide better estimates of thermal profiles than spot measurements taken a few times a day (Taylor et al., 2004); however, we cannot rule out the possibility that differences in animal condition or lags resulting from differences in method of measurement might have contributed. If lower temperatures are selected by skinks in response to potential dehydration in the wind, skinks may maintain a similar mean absolute deviation to those not exposed to wind as a compensatory response to maximise thermal gain while thermoregulating between cooler temperatures to minimise dehydration. The outcome of such a strategy would be that while mean *T*_sk_ was similar across all three treatments, skinks in the 0 m s^−1^ treatment were able to spend more time engaged in activities other than active thermoregulation. Increased time thermoregulating to compensate for lower *T*_b_ has been recorded in the lizard *Zootoca vivipara*; individuals living higher on an altitudinal gradient, where available operative temperatures were lower, compensated by spending 50% more time thermoregulating (Gvoždík, 2002). The maintenance of similar mean temperatures across treatments by McCann's skink, however, differs from studies that have recorded lizards selecting cooler temperatures when dehydrated (Le Galliard et al., 2021; Sannolo and Carretero, 2019). This difference may be due to skinks in this experiment thermoregulating in a way that anticipated a risk of dehydration while not necessarily becoming significantly dehydrated over the duration of the test. Such an adaptive response is plausible, as the skinks used in this experiment were from a sub-alpine site which is exposed and relatively windy. Nevertheless, the fact that thermoregulatory strategy differed among treatments and that lower selected *T*_b_ values were potentially compensated by increased time spent thermoregulating upholds the assumptions of the optimality model of thermoregulation (Huey and Slatkin, 1976).

In addition to changes in mean thermoregulatory strategy in response to increasing costs of thermoregulation, there may also be differences among individuals in how they incorporate these strategies. Interestingly, our results suggest that some of the variance among treatments for some measurements was due to individual differences in thermoregulation strategies that differed among skinks but were consistent across treatments. This effect was also observed here with the identity of the skink accounting for some of the observed variance in the amount of time spent thermoregulating, the number of shuttling events, mean *T*_sk_, mean absolute deviation and TPR. A similar result was reported for *T. torquatus* in that while the optimal amount of thermoregulation varied among individuals, different individuals made different thermoregulatory decisions under similar conditions (Leirião et al., 2019). In other words, there is evidence that thermoregulatory ‘personalities’ exist within skinks that are evident across treatments. Our results add to a small but growing number of studies indicating that thermal types within populations (i.e. personality) have measurable effects on thermoregulation in lizards (Michelangeli et al., 2018; Stapley, 2006).

In the field, a previous study found that strong winds (measured qualitatively) strongly affected the ability of McCann's skinks to raise their *T*_b_ above *T*_a_ (Hare et al., 2009). Similarly, field studies of other lizards have found that *T*_b_ is negatively correlated with windspeed (Maia-Carneiro et al., 2012; Ortega et al., 2017; Gontijo et al., 2018). These results could reflect thermoregulation in a thermal environment where the availability of optimal operative temperatures is reduced by the convective cooling of wind. However, in light of our results here, lower field *T*_b_ might be due to lizards selecting lower body temperatures than they would in low-wind conditions, especially if *T*_b_ is recorded as a spot temperature (which may not capture the full breadth of the range of thermoregulation in the way that our bio-loggers allowed). Patterns of emergence in the field in windy conditions of both McCann's skink and other species of dry-skinned ectotherms also do not align with the results found here. In the field, windspeed has been negatively correlated with emergence of dry-skinned ectotherms, including McCann's skink and two larger species in the same genus (Coddington and Cree, 1997; Hare et al., 2009; Logan et al., 2015; Sun et al., 2001). We hypothesise that in our study, a risk of eventual dehydration modulates thermoregulation under circumstances where the probable rate of dehydration was low. In the field, access to free water may be limited and the hydration of lizards may be poorer, eventually making the benefits of engaging in thermoregulation at all too great a risk.

To our knowledge, no previous experiments have attempted to measure the effects of controlled windspeeds on the behavioural thermoregulation of an ectotherm. Our results indicate that the physiological costs of exposure to wind, namely dehydration, may lead to lizards selecting lower body temperatures even when available operative temperatures are similar. Global climate heating poses specific risks to lizards; it has been estimated that increasing *T*_a_ alone could lead to the extinction of up to 20% of lizard species before 2080 (Sinervo et al., 2010; but see Clusella-Trullas and Chown, 2011). Changes in wind patterns and characteristics due to climate heating may therefore result in shifts in associated ecological costs or benefits. More frequent reductions in the set-point range due to wind may lead to more time being spent thermoregulating, leaving less time available for foraging, maintaining territories or other important ecological activities. Wind, therefore, and how it changes with climate warming may be a critical, but currently understudied, factor in determining the impact of climate warming on lizard species.

## Supplementary Material

Supplementary information
