# Supplementary material for: Wind of change: a diurnal skink thermoregulates between cooler set-points and for an increased amount of time in the presence of wind
Source: J Exp Biol. 2022 Mar 30;225(6):jeb244038. doi: 10.1242/jeb.244038 (PMC9001919; doi:10.1242/jeb.244038)
Supplement: Supplementary information [file jexbio-225-244038-s1.pdf]

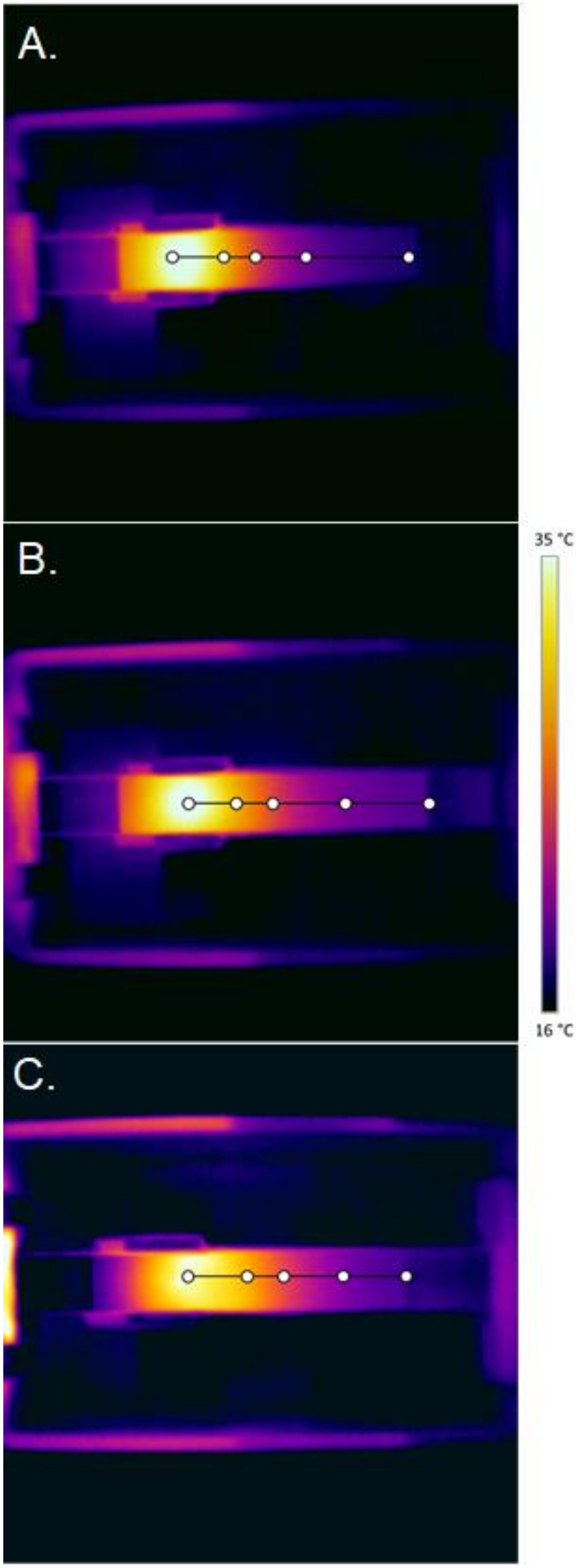

Journal of Experimental Biology • Supplementary information

Fig. S1. Thermograms of the experimental apparatus during the 0 m/s tests (A), 2 m/s tests (B) and 6 m/s tests (C) at steady state temperatures. Thermograms were taken with a FLIR i60 infrared camera and processed using FLIR Tools v6.4 (FLIR Systems, Wilsonville, USA). Emissivity is set to 0.95 and the reflected temperature was set to 16 °C (the ambient temperature of the room). Colour distribution is temperature linear. The white circles annotating each thermogram represent 35 °C, 30 °C, 25 °C, 20 °C and 18 °C points from left to right. The inset table shows the relative distances between these points for each thermogram. The scale bar applies to all three panels.

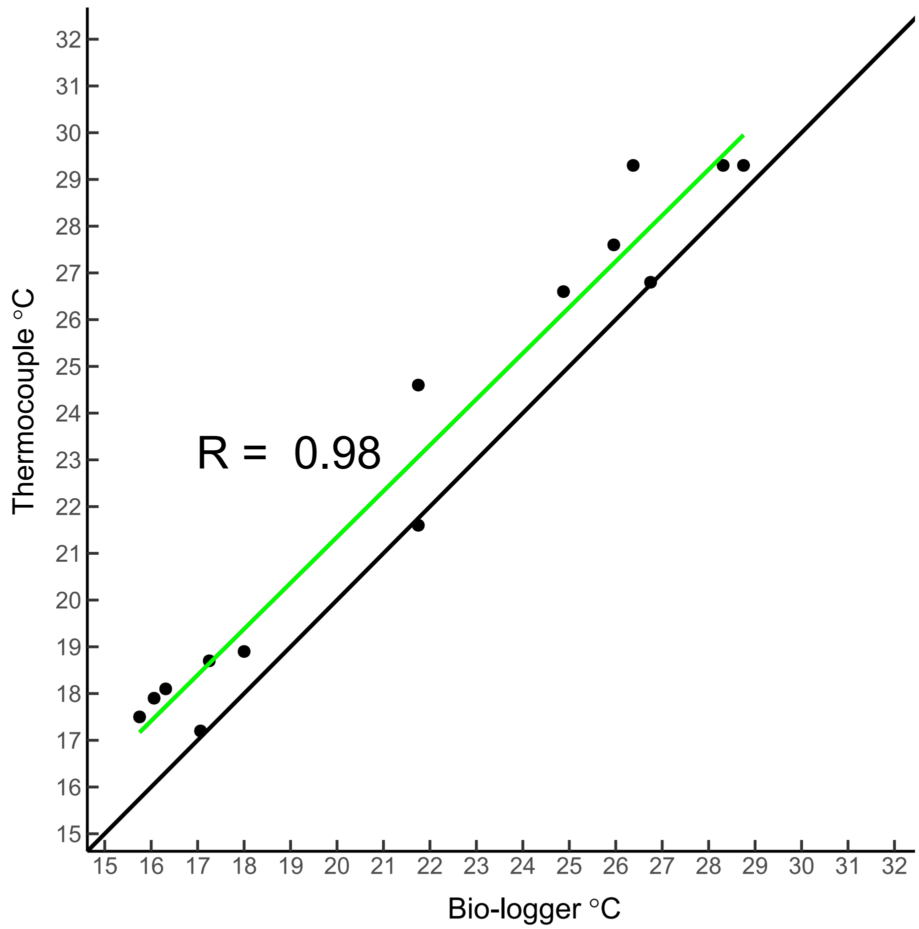

Fig. S2. Skink skin temperatures and cloacal temperatures of 14 skinks measured using a dorsally placed bio-logger and a thermocouple respectively. The green line is a linear trendline (*P* <0.01) and the dashed black line is a 1:1 identity line.

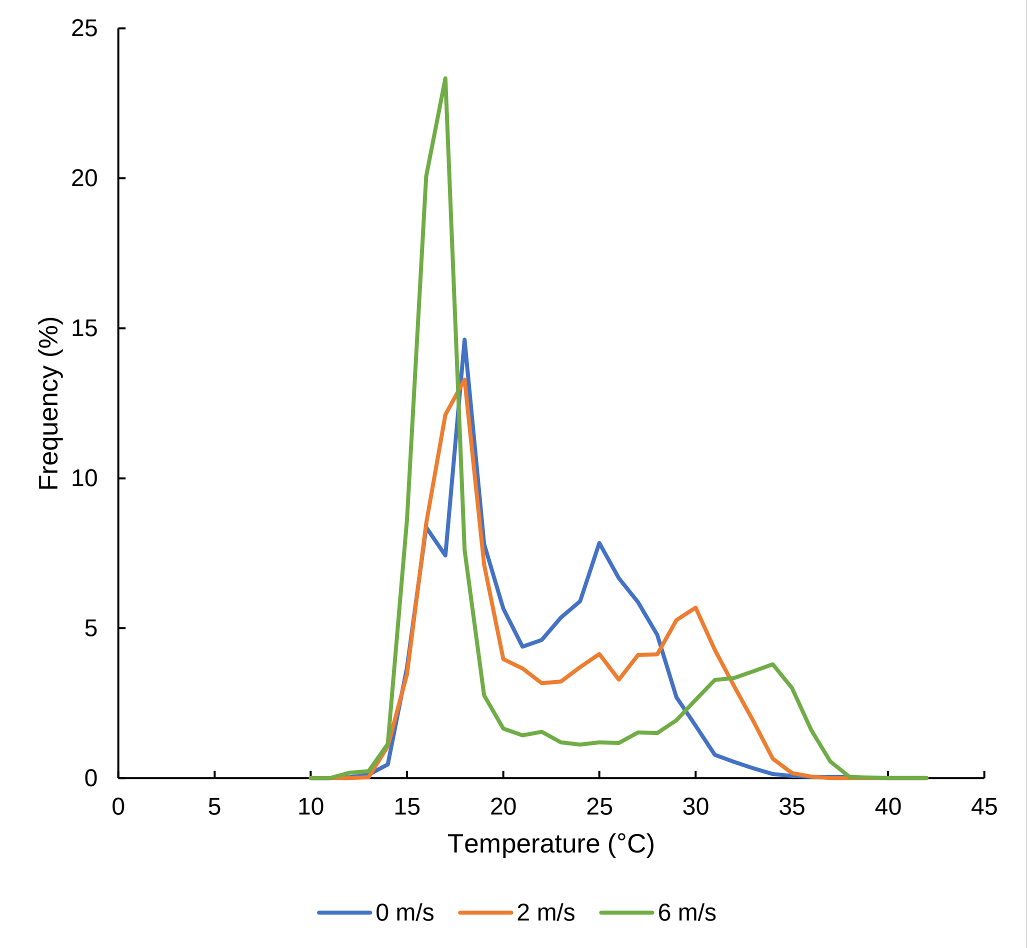

Fig. S3. Frequency of skink skin temperatures recorded over a 90-minute period by a dorsally placed bio-logger for three treatments: 0 m/s, 2 m/s, and 6 m/s (*n*=17, 23, 23).

Table S1. Distances between annotated points shown in Figure S1 and the total length of the temperature gradient for the three experimental treatments.

	A (0 m/s)	B (2 m/s)	C (6 m/s)
**35 °C – 30 °C**	53 mm	49 mm	56 mm
**30 °C – 25 °C**	32 mm	37 mm	35 mm
**25 °C – 20 °C**	50 mm	67 mm	58 mm
**20 °C – 18 °C**	105 mm	86 mm	62 mm
**Total length**	240 mm	239 mm	211 mm
